# Internet Communication Disorder and the structure of the human brain: initial insights on WeChat addiction

**DOI:** 10.1038/s41598-018-19904-y

**Published:** 2018-02-01

**Authors:** Christian Montag, Zhiying Zhao, Cornelia Sindermann, Lei Xu, Meina Fu, Jialin Li, Xiaoxiao Zheng, Keshuang Li, Keith M. Kendrick, Jing Dai, Benjamin Becker

**Affiliations:** 1 The Clinical Hospital of Chengdu Brain Science Institute, MOE Key Laboratory for Neuroinformation, University of Electronic Science and Technology of China, Chengdu, China; 2 Institute of Psychology and Education, Ulm University, Ulm, Germany; 3 Chengdu Mental Health Center, Chengdu, 610031 China

## Abstract

WeChat represents one of the most popular smartphone-based applications for communication. Although the application provides several useful features that simplify daily life, a growing number of users spend excessive amounts of time on the application. This may lead to interferences with everyday life and even to addictive patterns of use. In the context of the ongoing discussion on Internet Communication Disorder (ICD), the present study aimed to better characterize the addictive potential of communication applications, using WeChat as an example, by examining associations between individual variations in tendencies towards WeChat addiction and brain structural variations in fronto-striatal-limbic brain regions. To this end levels of addictive tendencies, frequency of use and structural MRI data were assessed in n = 61 healthy participants. Higher tendencies towards WeChat addiction were associated with smaller gray matter volumes of the subgenual anterior cingulate cortex, a key region for monitoring and regulatory control in neural networks underlying addictive behaviors. Moreover, a higher frequency of the paying function was associated with smaller nucleus accumbens volumes. Findings were robust after controlling for levels of anxiety and depression. The present results are in line with previous findings in substance and behavioral addictions, and suggest a similar neurobiological basis in ICD.

## Introduction

In the first quarter of 2017 49.7% of the world population had access to the Internet. In the country with the largest population today, China, 52.7% of the population have been online in 2017^1^. The Internet is increasingly accessed via mobile devices, particularly smartphones. Penetration rates of smartphones are growing at incredible speed with currently 663 million smartphone users alone in China^2^. Smartphone applications simplify daily life and promote social interactions via social network and messenger platforms, however, a number of users develop excessive, or even addictive, patterns of smartphone use that interfere with daily life and mental health^3–6^.

The importance of studying the neural basis of addictive Internet use has been recently emphasized by the inclusion of Internet Gaming Disorder (IGD) as an emerging mental disorder in the DSM-5^7^. IGD represents a specific form of Internet addiction^8^, characterized by a loss of control of use, development of tolerance and loss of interest in alternative activities^7,9^. The symptoms strongly resemble dysregulations observed in other addictive disorders, including substance and behavioral addictions. During the last 20 years (e.g. early work by Young^10,11^) elaborate conceptualizations have been developed to describe mechanisms of Internet addiction that provide a research framework for specific forms of Internet addiction (e.g. IGD), as well as broader forms such as Internet Communication Disorder referring to addictive use of social network and messenger platforms^12^. With respect to the neurobiological basis of Internet addiction, converging evidence points to dysfunctions in the fronto-striatal-limbic circuitries accompanied by impaired executive functions, maladaptive reward processing and deficient emotion regulation^12–14^. This pattern partly converges with pathological alterations that have been documented in substance addiction and pathological gambling^15–19^, suggesting that disruptions in the fronto-striatal-limbic circuitries represent a common pathological denominator for addictive disorders that mirror exaggerated reward sensitivity and impulsivity in the amygdala-striatal systems in the context of impaired regulatory control via frontal regions.

The smartphone is a relatively new technical achievement and research has only recently begun to evaluate its addictive potential^20–22^ (for a direct comparison of Internet and smartphone addiction see the here cited articles^21^). A recent study that tracked smartphone use in everyday life revealed usage times in the range of about 2.5 h per day, suggesting that smartphones increasingly dominate daily life^23^. Moreover, this study demonstrated that in particular smartphone-based social communication applications such as WhatsApp (>one billion users worldwide^24^) represent driving forces of excessive smartphone usage. Initial findings suggest that escalating use of these applications may be rooted in acute and long term effects on the fronto-striatal-limbic circuitry, possibly reflecting their addictive potential. Accumulating evidence suggests that the use of these applications is accompanied by increased ventral striatal activity^25–27^ possibly reflecting the contribution of reward- and reinforcement-related mechanisms to the development of addictive patterns of use. Initial studies on the long term effects of excessive use suggest detrimental effects on emotional and cognitive functions that depend on the integrity of the fronto-striatal-limbic circuitry, including increased levels of depression and anxiety^28^, deficient inhibitory control^29^ and enhanced distraction via smartphones^30–32^. Notably, a recent prospective study provided initial evidence for a causal link between escalating smartphone use and increasing impulsivity as well as a declining cognitive and social functioning^33^. Studies employing structural brain imaging revealed initial evidence for an association between escalating social media use and decreased volumes of the nucleus accumbens (NAc)^34,35^ (however, see He *et al*.^36^), a key reward processing node in the ventral striatum, and the amygdala^35,36^ (however, see Montag *et al*.^34^). Smaller volumes of these regions have been previously associated with the development and maintenance of substance addiction^19,37,38^, with preclinical data emphasizing an important contribution of neuroplastic changes in these regions to pathological changes in motivational, impulsive and habitual behaviors that drive addiction^39–42^. Findings with regard to regulatory control regions, however, remained inconsistent, and one study even reported that (dorsal) anterior cingulate (ACC) volume increased as a function of social network addiction^35^. This finding contrasts with the important role of the ACC in implementing frontal control over limbic-striatal regions, and previous reports suggesting an association between decreased volumes of this region and inhibitory control deficits in substance and Internet gaming addicted populations^43–46^.

In summary, some of the here reviewed studies revealed initial evidence for ventral striatal and amygdala morphological pecularities in (excessive) social media users, possibly reflecting that the addictive potential of these applications is based on shared mechanisms with other addictions. In contrast, findings on the ACC remained inconsistent^35,36,47^ and might reflect a distinct characteristic of Internet Communication Disorder. However, conclusions are hampered by methodological limitations of the initial studies, particularly the small sample sizes and lack of control variables, as well as by the functional and anatomical heterogeneity of the ACC^48–50^, with converging evidence suggesting a division into dorsal (caudodorsal) and ventral (subgenual, pregenual) subregions^48,51,52^. In line with their distinct anatomical and functional connections^48,53^, dorsal regions are predominantly engaged in cognitive and motor functions whereas ventral regions contribute to emotion regulation and behavioral control^52,54^. Indeed, studies in patients with mental disorders emphasize the importance of subregion-specific anatomical changes of the ACC in disorders characterized by deficient cognitive and emotional functioning, such as depression and anxiety disorders^55–57^.

Within the context of a dimensional conceptualization of mental disorders^58^ the present study examined associations between levels of addictive social media/messenger use and morphological variations in the NAc, amygdala and ACC in a sample of healthy subjects (for a similar approach see Luo *et al*.^59^). To specifically clarify inconsistencies regarding the ACC, subregion-specific gray matter volumes of the major ACC subdivisions were assessed in a comparably large samples (n = 61). Given that elevated levels of depression and anxiety have been related to both, excessive social media use^28^ and fronto-striatal-limbic morphology^60–62^, levels of depression and anxiety were controlled for.

Whereas previous studies on brain structural alterations associated with excessive social media/messenger use in European or American populations and focused on Facebook/general social network usage^34–36^, the present study was conducted in China where WeChat (; Wēixìn, “micro message”, currently >900 million users^63^) represents one of the most popular social communication platforms. The literal translation of Wēixìn (“micro message”), refers to the initial development of WeChat as mobile messaging application that allows to send short text messages to individuals or groups and share photos or other files. Whereas the early versions of the Chinese WeChat were thus similar to Western messaging applications like WhatsApp, more recent versions integrate numerous additional functions and platforms including social sharing, paying, banking, and city services like paying traffic fines and booking transportation. Using this multi-purpose integration WeChat has penetrated several aspects of daily life and has become one of the largest social media platforms worldwide in terms of monthly active users.

To determine individual levels of addictive behavior in the context of WeChat usage a validated digital addiction scale (short-Internet addiction scale; s-IAT^64^) that assesses key diagnostic criteria, namely loss of control/time management and craving/social problems, was adopted. Given that previous research has associated the frequency of online media use with both, levels of addictive symptoms^6,65,66^ and decreased NAc volumes^34^, the frequency of use of the most popular WeChat functions (texting, voice messaging, paying) was additionally assessed. To determine associations between individual variations in the level of addictive symptoms and the frequency of use with brain structural variations, T1-weigthed Magnetic Resonance Imaging (MRI) data was acquired from all subjects.

**Figure 1:**
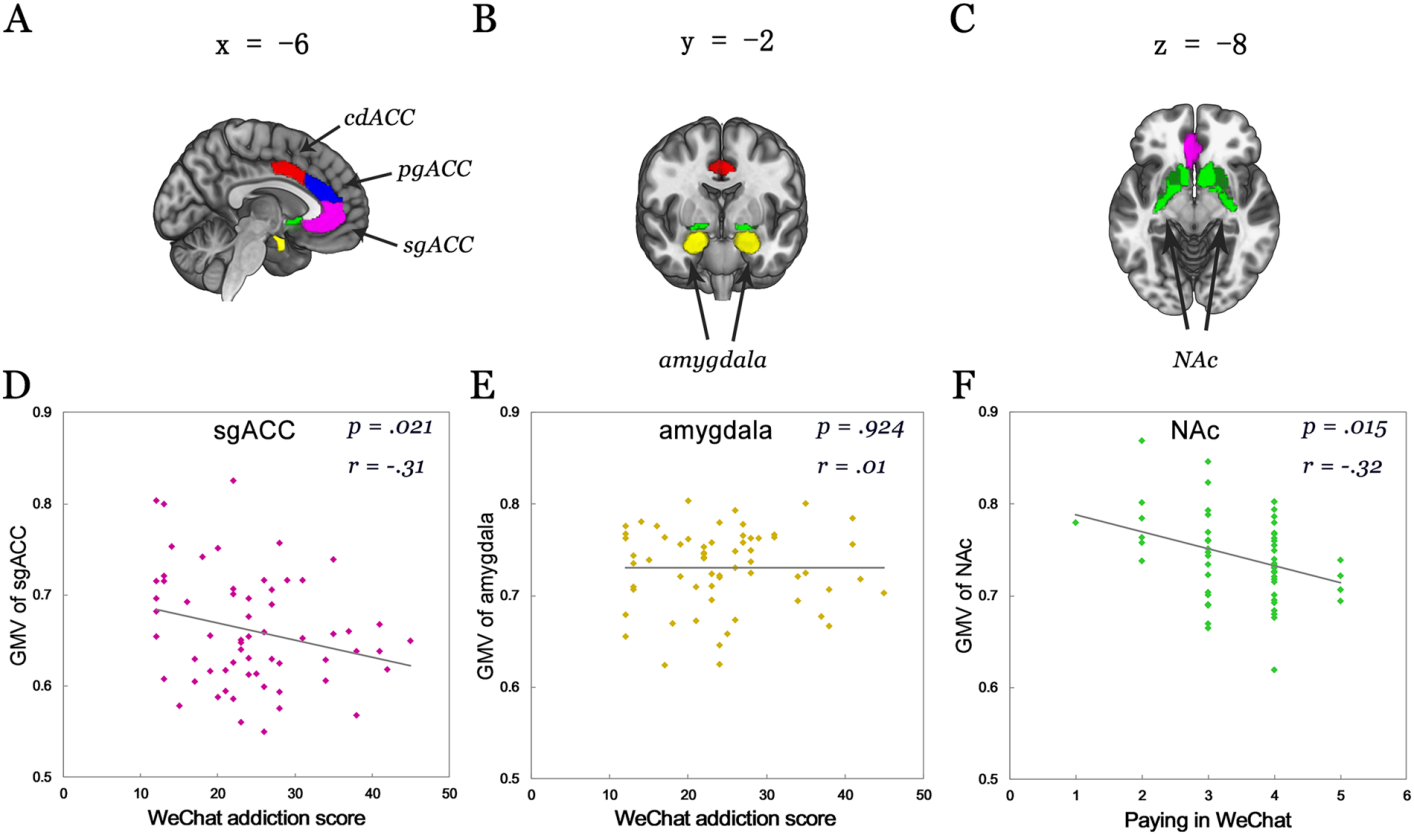
The regions of interest examined in the present study and associations between gray matter volume with WeChat addiction scores and use frequency. **(A–C)** Displays the regions examined in Montreal Neurological Institute (MNI) space. (**D–E**) Displays the correlations between brain volumes and WeChat addiction scores (**D,E**), and the frequency of use of the paying function **(F)** in the n = 61 participants. P and r values displayed correspond to correlation analysis (two-tailed) including age, gender, depression and anxiety included as control variables.

Based on previous studies^34–36^, we expected that higher levels of addictive symptoms associate with decreased gray matter volume (GMV) of the NAc and amygdala. In the context of the important role of deficient regulatory control in addictive disorders and initial behavioral evidence for associations between excessive smartphone use and deficits in this domain^29,33^, we expected a negative association between levels of addictive symptoms and GMV in ventral subregions of the ACC. Given inconsistent findings with regard to the involvement of the dorsal ACC (dACC) in social media addiction^35,36^ no directed hypothesis with respect to this region was specified. Finally, in line with our previous research^34^ we expected that a higher frequency of use would associate with decreased NAc volumes. An overview of the regions examined is provided in Fig. 1A–C.

## Results

### Descriptive statistics

Table 1 provides descriptive statistics on the WeChat addiction scores and smartphone/WeChat usage variables (details on the scales are given in the Methods section).

**Table 1:** Descriptive statistics of smartphone variables including WeChat usage.

Investigated Variables	Mean and Standard Deviation; Actual Range
Smartphone use in hours each week for private/leisure	M = 24.28, SD = 18.48; actual range: 2–100
Smartphone use in hours each week for business	M = 7.85, SD = 6.68; actual range: 0–30
Smartphone usage in hours each week for both leisure and business (added from the previous two items)	M = 32.13, SD = 22.03; actual range: 3–120
Smartphone Addiction (possible range: 10–60)	M = 34.43, SD = 9.01; actual range: 14–52
WeChat use in hours each week for private/leisure	M = 9.95, SD = 10.30; actual range: 1–50
WeChat use in hours each week for business	M = 3.85, SD = 6.63; actual range: 0–40
WeChat usage in hours each week for both leisure and business (added from the previous two items)	M = 13.80, SD = 14.22; actual range: 1–70
WeChat Addiction (possible range: 12–60)	M = 24.33, SD = 8.44; actual range: 12–45
WeChat Addiction Loss of Control (possible range: 6–30)	M = 12.64, SD = 4.49; actual range: 6–23
WeChat Addiction Social Problems (possible range: 6–30)	M = 11.69, SD = 4.21; actual range: 6–22
WeChat: Texting (possible range: 1–5)	M = 3.97, SD = 0.97; actual range: 2–5
WeChat: Voice Messaging (possible range: 1–5)	M = 2.67, SD = 0.96; actual range: 1–5
WeChat: Paying in the store (possible range: 1–5)	M = 3.54, SD = 0.85; actual range: 1–5

### WeChat addiction and distinct usage of WeChat channels

Table 2 presents the correlation between WeChat addiction scores and usage of its distinct functions. Given the significant correlation of WeChat addiction scores as well as sending text messages with age (e. g. r = 0.27, p = 0.035 for the total WeChat addiction scale; r = 0.31, p = 0.016 for sending text messages), Table 2 presents partial correlations (of note, as there were no gender differences in the WeChat addiction scale or the usage of distinct functions, gender was not included in these analyses). A hierarchical stepwise regression model with age and gender inserted in a first block and all distinct WeChat functions in a second step yielded a significant model with age (7.3% explained variance) and sending voice messages (22.3% additional explained variance) as the most important predictors for WeChat addiction scores. These variables together explain 29.6% in the variation of WeChat addiction scores (F(2, 58) = 12.17, p < 0.001).

**Table 2:** Partial correlation between WeChat addiction and distinct usage of WeChat channels.

	WeChat	WeChat LC	WeChat SP	Texting	Voice	Paying
WeChat		r = 0.97, p < 0.001	r = 0.97, p < 0.001	r = 0.25, p = 0.054	r = 0.49, p < 0.001	r = 0.25, p = 0.058
WeChat LC			r = 0.87, p < 0.001	r = 0.23, p = 0.079	r = 0.49, p < 0.001	r = 0.25, p = 0.057
WeChat SP				r = 0.26, p = 0.048	r = 0.46, p < 0.001	r = 0.23, p = 0.080
Texting					r = 0.21, p = 0.112	r = 0.41, p = 0.001
Voice						r = 0.16, p = 0.227
Paying						

WeChat LC: Loss of Control sub-scale; WeChat SP: Social Problems sub-scale. Partial correlations are calculated with age included as controlled variable.

### Correlations between brain structure and levels of WeChat addiction

A negative correlation was observed between WeChat addiction scores and the GMV of the subgenual ACC (sgACC) while controlling for age and gender (r = −0.26, p = 0.043) (see Fig. 1D). Findings remained stable after controlling for depression and anxiety (r = −0.31, p = 0.021). No significant associations between WeChat addiction scores and the more dorsal regions of the ACC were observed (pregenual ACC, pgACC, r = 0.01, p = 0.942; caudodorsal, cdACC, r = 0.12, p = 0.381 controlling for age and gender; both p > 0.726 after additionally controlling for anxiety and depression). No associations were observed between WeChat addiction scores and GMV of the NAc (r = −0.07, p = 0.601 controlling for age and gender, r = −0.074, p = 0.587 after additionally controlling for anxiety and depression) or the amygdala (r = 0.05, p = 0.702 controlling for age and gender, p = 0.924 after additionally controlling for anxiety and depression; see also Fig. 1E).

### Associations with WeChat usage parameters

Given that our previous study on Facebook usage specifically identified a negative association between GMV of the NAc and behavioral indices of excessive smartphone-based social media usage (daily frequency of Facebook checking/duration of Facebook usage on smartphone^34^), an additional analysis specifically examined associations between WeChat usage behavior and GMV of the NAc. In line with the previous results^34^ a negative correlation was observed between lower GMV of the NAc and higher usage of the application, although in this case specifically for paying functions (r = −0.29, p = 0.029, controlled for age and gender; r = −0.32, p = 0.015 with anxiety and depression additionally controlled for; non-parametric analysis using Spearman correlation; rho = −0.29, p = 0.022) (see Fig. 1F). Neither the total WeChat addiction scale, voice messaging nor texting showed significant associations with NAc GMV (all p > 0.183, two-tailed, controlled for age, gender; all p > 0.098, two-tailed, controlled for age, gender, anxiety and depression; non-parametric analyses using Spearman correlation; all p > 0.114).

## Discussion

The present study aimed to investigate associations between individual variations in the levels of WeChat addiction with gray matter volumes of the amygdala, NAc and and subregions of the ACC. Based on previous findings associations between usage frequency and NAc volume was additionally examined. Higher levels of self-reported addictive symptoms and more frequent usage (of the paying service) were associated with lower gray matter volumes in the ventral (subgenual) ACC and NAc, respectively. A similar relationship between higher levels of addictive symptoms or more excessive patterns of use and volume reductions in these regions has been repeatedly reported in substance addiction^44^ as well as behavioral addictions including generalized Internet addiction^47^ and Internet gaming disorder^67,68^. Together with previous research on the brain structural correlates of excessive social media and messenger use^34–36^ the present findings suggest that structural alterations in the fronto-striatal-limbic circuitry represent a common denominator across different types of digital addiction, including Internet Communication Disorder.

Based on inconsistent findings with regard to structural changes of the ACC in Internet Communication Disorder, the present study specifically explored associations between levels of WeChat addiction symptoms and ACC volume on a subregional level. This approach revealed that higher symptom levels were associated with lower volume of the ventral ACC, specifically the sgACC. In contrast to a previous study reporting that dACC volumes increased as a function of higher social network addiction^35^, the present study did not observe significant associations with the dACC region. These inconsistencies may be due to a higher sensitivity of the subregional approach, differences in the levels of addictive symptoms in the samples examined (some participants scoring 40–45 points on the scale with a maximum of 60 points in the present sample), sample size (n = 61 in the present study vs. n = 20 in the He *et al*. study^35^), or the use of different scales to assess individual variations in social media addiction (with the present study focusing on WeChat addiction rather than general social media addiction).

The present findings of reduced sgACC volume in the context of higher addictive symptom scores converge with previous findings on reduced ACC volumes in other addictive disorders, including substance addiction^44^ and Internet Gaming Disorder^67,68^. In line with the regulatory role of the ACC, reduced volumes of this region in Internet addiction have been associated with deficient cognitive control^68^ and increased impulsivity^46^, and thus might contribute to the loss of behavioral and emotional control which represents a key symptom across addictive disorders. Given that convergent evidence from previous research using different methods including electroencephalography (EEG) and MRI indicates an important role of ACC dysfunctions in the development and maintenance of excessive and addictive digital media use (for an overview see Montag, Duke & Reuter^69^), the present findings argue for similar disruptions in frontal control regions across different forms of digital addiction.

On the subregional level the present analysis approach demonstrated a specific association with volumes of the sgACC. The sgACC projects to limbic and striatal regions including the NAc^53^ and plays a pivotal role in implicit emotion regulation^54,70^ and inhibitory control^71^. The present findings thus converge with previous conceptualizations discussing enhanced impulsivity and impaired inhibitory control as endophenotype markers across psychiatric diagnoses, that precede the development of the complete clinical picture of the disorder^58^.

Further support for the functional relevance of reduced sgACC volumes in this behavioral domain comes from previous morphological studies reporting associations between decreased sgACC volumes with deficient emotional and inhibitory control, including increased subjective experience of chronic stress^72^, reduced behavioral control during acute stress^68^, behavioral disinhibition in dementia patients^73^ as well as lower resilience in the normal population^74^. These functional characterizations of reduced sgACC volumes suggest that the present observations might either reflect that increasing levels of ICD symptoms are accompanied by a loss of regulatory control (as suggested by initial prospective data^33^), or represent a pre-existing vulnerability factor, such as a generally reduced inhibitory control or resilience, increasing the risk for the development of addictive behavior.

Moreover, in line with previous studies^34,36^ an association between a more frequent usage of a social media platform and smaller NAc volumes was observed, emphasizing the importance of alterations in the ventral striatal reward system in ICD. Alterations in ventral striatal morphology have been repeatedly observed in related disorders such as Internet Gaming Disorder possibly reflecting adaptations in the striatal reward system^43^. Although previous studies predominantly reported decreased ventral striatal volume in excessive Internet gaming^43^, increased volumes^75,76^ have also been demonstrated. As discussed in the context of the present findings on the ACC, the association between lower gray matter volume of the NAc and higher usage of WeChat’s paying function argues for similar neurobiological mechanisms across different addictive disorders. Mounting evidence from research on alcohol and nicotine addiction demonstrates similar associations between lower gray matter volume of the NAc and higher levels of addictive symptoms^77,78^. Reduced NAc volumes have additionally been observed in heroin addicts^79^. A noteworthy finding in our study is a significant association between gray matter volumes of the NAc and WeChat related variables specifically observed for paying, but not for functions such as texting or voice messaging (although associations with texting showed a similar trend for negative associations, see Table S3). In contrast, no associations were observed between NAc volume and the WeChat addiction scores. Together, this demonstrates the importance for a differential examination of excessive usage and problematic (addictive) behavior as well as of different functions of the applications to delineate a fine-grained picture of the underlying biological mechanisms that promote excessive and potentially addictive social media usage.

The present findings need to be interpreted in the context of some limitations. First, although the examination of different ACC subregions facilitated a more sensitive analysis of this heterogeneous structure, the approach increased the number of regions examined and the significance level was not adopted for the number of regions of interest. Second, the present study did not assess general tendencies towards social media overuse. Although such a scale might be highly correlated with WeChat addiction tendencies (see also correlations with smartphone addiction in the supplementary material), the brain structure associations might have been different in the present data set. Third, we relied on a rather young (Asian) student sample. Therefore, it is not clear if the present results can be generalized to the broader population of WeChat users going also beyond Asian populations. Moreover, future studies should consider implementation of smartphone tracking technologies from Psychoinformatics^80^ accompanying self-report assessment as used in previous research^23,34,81,82^. Given the overlap of the here reported WeChat-brain structure associations with other reported findings in the literature, we are still optimistic that this might be the case. Finally, the present data set only investigates structural associations in the human brain and provides no insights into functional aspects in the context of social media use. Future studies should aim to characterize alterations on the functional level.

In summary, these initial findings on the brain structural substrates of addictive behavior related to WeChat show that in line with previous reports and overarching neurobiological conceptualizations in substance-based and behavioral addictions including Internet addiction, lower ACC gray matter volumes are associated with higher addictive tendencies towards using this smartphone-based platform. Moreover, the present results provide further evidence for the important role of the NAc in excessive digital (over-)use.

## Methods

### Participants and Protocols

N = 67 healthy university students were recruited for the present study. Inclusion criteria were (1) no previous or current neurologic/mental disorder according to self-report (2) no contraindications for magnetic resonance imaging (MRI) (e.g. metal implants, pregnancy) (3) ownership of a smartphone and use of the WeChat application. In addition, depression and anxiety scores were collected from the participants and these resulted in non-obstrusive scores (depression score from BDI-II^83^, mean = 8.37, SD = 6.64; trait anxiety from STAI^84^, mean = 42.27, SD = 8.34; numbers are presented for the final sample of N = 61 participants). Based on the study inclusion criteria (n = 1 non using WeChat) and MRI data quality assessments (data from n = 5 participants excluded) 61 participants entered the final analyses (40 males and 21 females; mean-age: 22.34; SD = 2.29). WeChat addiction scores and usage behavior were assessed using self-report questionnaires (details see questionnaires). To determine associations between individual variations in the level of addictive symptoms and WeChat usage with brain structural variations, T1-weigthed MRI data was acquired from all subjects.

The study and protocols were approved by the local ethic committee of the University of Electronic Science and Technology (UESTC), Chengdu, China and participants provided informed consent before study inclusion. The study and procedures were in line with the most recent version of the Declaration of Helsinki.

### Questionnaires

All participants were administered a modified version of the validated short-Internet addiction scale (s-IAT) as provided by Pawlikowski *et al*.^64^ that – in line with the proposed diagnostic criteria for Internet addiction - assesses loss of control/time management and craving/social problems as key characteristics of Internet addiction. In order to specifically assess levels of addictive WeChat use we carefully adopted the wordings with respect to WeChat usage. E. g. the original item called “How often do you find that you stay on-line longer than you intended?” has been changed to “How often do you find that you stay longer on WeChat than you intended?” (for a complete list of the items see Table 3). Participants answered the twelve items using a five point Likert scale ranging from never (1) to very often (5). Internal consistencies of the questionnaire were excellent (Cronbach’s α, complete scale, α = 0.93; loss of control/time management subscale (items 1, 2, 3, 6, 8, 9), α = 0.90; craving/social problems subscale (items 4, 5, 7, 10, 11, 12), α = 0.83). We report only associations with the total scale in the present work, given that results regarding our main hypothesis on the ventral ACC were equally strong for both subscales (association with sgACC: r = −0.29, p = 0.033 for loss of control and r = −0.31, p = 0.020 for social problems when controlling for age, gender, anxiety and depression).

**Table 3:** Chinese and English version of WeChat addiction test (**-**AT; WC-AT). Note that this is a modified version of the s-IAT as presented in Pawlikowski *et al*.^64^.

1、? (LC)
2、? (LC)
3、? (LC)
4、? (SP)
5、? (SP)
6、? (LC)
7、? (SP)
8、? (LC)
9、? (LC)
10、? (SP)
11、? (SP)
12、? (SP)
Answer options: (1), (2), (3), (4), (5); LC: loss of control/time management; SP: craving/social problems
1. How often do you find that you stay on WeChat longer than you intended? (LC)
2. How often do you neglect household chores to spend more time on WeChat? (LC)
3. How often do your grades or school work suffer because of the amount of time you spend on WeChat? (LC)
4. How often do you become defensive or secretive when anyone asks you what you do on WeChat? (SP)
5. How often do you snap, yell, or act annoyed if someone bothers you while you are on WeChat? (SP)
6. How often do you lose sleep due to being on WeChat late at night? (LC)
7. How often do you feel preoccupied with WeChat when off-line, or fantasize about being on WeChat? (SP)
8. How often do you find yourself saying “just a few more minutes” when on WeChat? (LC)
9. How often do you try to cut down the amount of time you spend on WeChat and fail? (LC)
10. How often do you try to hide how long you’ve been on WeChat? (SP)
11. How often do you choose to spend more time on WeChat over going out with others? (SP)
12. How often do you feel depressed, moody, or nervous when you are off-line, which goes away once you are back on WeChat? (SP)

Answer options: (1) never, (2) rarely, (3) sometimes, (4) often, (5) very often; LC: loss of control/time management; SP: craving/social problems.

WeChat usage behavior was further characterized by administering a questionnaire that assessed hours per week for private and business matters, the frequency of use of texting, voice-messaging and paying with the application (details see Table 4).

**Table 4:** Chinese and English version asking for amount of WeChat use and its distinct functions.

: ________
:________
:



Answer options: (1), (2), (3), (4), (5)
WeChat use in hours each week for private/leisure: ______
WeChat use in hours each week for business: ______
Please indicate how often you use the following functions:
Texting
Voice-messaging
Paying in the store

Answer options: (1) never, (2) rarely, (3) sometimes, (4) often, (5) very often.

Given that WeChat is nearly exclusively used on mobile devices, smartphone addiction was additionally assessed using a validated questionnaire provided by Kwon *et al*.^20^. This short questionnaire consisting of 10 items with answer option 1–6 (1 = strongly disagree to 6 = strongly agree) assesses individual differences in smartphone addiction including items targeting the main characteristics of addictive usage such as preoccupation with the smartphone and loss of control. Internal consistency of the smartphone addiction scale was excellent (α = 0.83). This questionnaire was not significantly associated with brain structure in the predefined regions of interest. Therefore, corresponding findings are presented in the supplementary material.

Based on accumulating evidence for associations between excessive smartphone use and increased levels of depression and anxiety^28^ and previous reports demonstrating associations between variations in levels of anxiety and depression and brain structure^60–62^, trait anxiety (TAI)^84^ and depressive symptoms (BDI-II)^83^ were assessed (Cronbach alpha was 0.64 for the Mandarin version of TAI^85^ and 0.86 for Mandarin version of BDI-II^86^). To control for potential confounding effects on associations between WeChat addiction and brain structure, analyses were recomputed including levels of anxiety and depression as additional covariates along with gender and age.

### Brain Structure: data acquisition and analysis

All participants underwent a T1-weighted brain structural assessment on a 3-Tesla GE MR750 system (General Electric Medical System, Milwaukee, WI, USA). Data was acquired using an FSPGR sequence with the following imaging parameters: repetition time = 5.97 ms, echo time = 1.97 ms, flip angle = 9°, field of view = 256*256 mm^2^, slice thickness = 1 mm, slice (coronal) number = 128, matrix = 256*256.

The structural data was preprocessed using the VBM8 toolbox as implemented in SPM12 (Statistical Parametric Mapping, http://www.fil.ion.ucl.ac.uk/spm/). The brain volumes were first segmented into different brain tissues including gray matter, white matter and CSF using the unified segmentation^87^ as implemented in SPM12 (“New Segment”) and subsequently non-linearly normalized to MNI space using the DARTEL algorithm^88^ while controlling for variances within individual total brain volumes by modulating the voxel values of the gray matter images with the warping parameters generated during affine transformation, which is proportional to total intracranial volume (TIV)^89^.

Quality assessment procedures included VBM toolbox algorithms that examine the individual covariance of each brain volume. In line with the recommendations for VBM analyses, images with covariance below 2 standard deviations from the mean (0.78) of the total data were excluded from further analysis (n = 5 participants). To minimize individual differences in spatial registration and increase signal-to-noise ratio (SNR), gray matter volumes were finally spatially smoothed with an 8 mm FWHM (full width at half maximum) kernel.

Based on our regional a priori hypotheses and in line with previous studies on social network use addiction^34–36^ the brain structural analyses focused on the anterior cingulate cortex (ACC), nucleus accumbens (NAc) and amygdala as a priori regions of interest (ROIs). To account for the functional heterogeneity of the ACC (Etkin *et al*.^52,54^) and in line with previous studies specifically targeting subregion-specific ACC engagement in mental disorders^55–57^ the subgenual ACC (sgACC), pregenual ACC (pgACC), and caudodorsal ACC (cdACC) was assessed. To determine associations with levels of WeChat addiction and usage, gray matter volumes (GMV) were extracted from bilateral masks derived from the Brainnetome atlas^90^ (see Fig. 1A–C for the ROIs examined in the present study).

First, descriptive statistics as well as the relations between WeChat addiction scores and usage of distinct WeChat functions are presented. Associations between individual variations in WeChat addiction and variations in regional GMV were examined using partial correlations (controlling for age and gender as well as age, gender, anxiety and depression; see introduction part). The frequency variables texting, paying and sending voice messages were non-normally distributed. However, given that skewness and kurtosis of the variables was <1, parametric partial correlations (controlling for age and gender as well as age, gender, anxiety and depression) were used to examine associations between individual variations in these variables and variations in NAc volume. To further validate the robustness of the associations between the frequency variables and NAc volume, results from Spearman’s nonparametric rank correlation are additionally reported. All analyses were performed using a two-sided p < 0.05 as significance threshold.

### Data Availability Statement

Upon reasonable request third parties will be granted access to the original data of this manuscript.

## Electronic supplementary material

Supplementary Information
