# Supplementary material for: Internet Communication Disorder and the structure of the human brain: initial insights on WeChat addiction
Source: Sci Rep. 2018 Feb 1;8:2155. doi: 10.1038/s41598-018-19904-y (PMC5794793; doi:10.1038/s41598-018-19904-y)
Supplement: Supplementary file 1 — Supplementary Information [file 41598_2018_19904_MOESM1_ESM.docx]

**Internet Communication Disorder and the structure of the human brain: initial insights on WeChat addiction (Supplementary Material)**

Christian Montag^1,2*+^, Zhiying Zhao^1*^, Cornelia Sindermann^2^, Lei Xu^1^, Meina Fu^1^, Jialin Li^1^, Xiaoxiao Zheng^1^, Keshuang Li^1^, Keith M. Kendrick^1^, Jing Dai^1,3^ Benjamin Becker^1+^

^1^The Clinical Hospital of Chengdu Brain Science Institute, MOE Key Laboratory for Neuroinformation, University of Electronic Science and Technology of China, Chengdu, China

^2^Institute of Psychology and Education, Ulm University, Ulm, Germany

^3^Chengdu Mental Health Center, Chengdu 610031, China

*the authors contributed equally

^+^correspondence: [christian.montag@uni-ulm.de](mailto:christian.montag@uni-ulm.de) / ben_becker@gmx.de

**Assessment of smartphone addiction**

All participants additionally completed a brief scale to assess smartphone addiction originally provided by Kwon et al. (2013). For the use in the present study the instrument was adopted. To give an example: Whereas Kwon et al. (2013, p. 5, Table 3) present the item „Missing planned work due to smartphone use“ we reworded this item to „I miss planned work due to smartphone use“. In our sample participants answered this questionnaire on a six point Likert scale ranging from 1=strongly disagree to 6=strongly agree, accordingly, higher scores reflect higher tendencies towards smartphone addiction. Technically the total questionnaire scores can range between 10 and 60. Internal consistencies as assessed by Cronbach’s α were excellent (α = .83). The questionnaire was complemented by two items asking for the time spend using the smartphone for leisure and business use (hours per week). The items are provided in **Table S1**.

**Table S1: Chinese and English version of the short smartphone addiction scale (modified from Kwon et al., 2013) – details see also method section in the main manuscript**


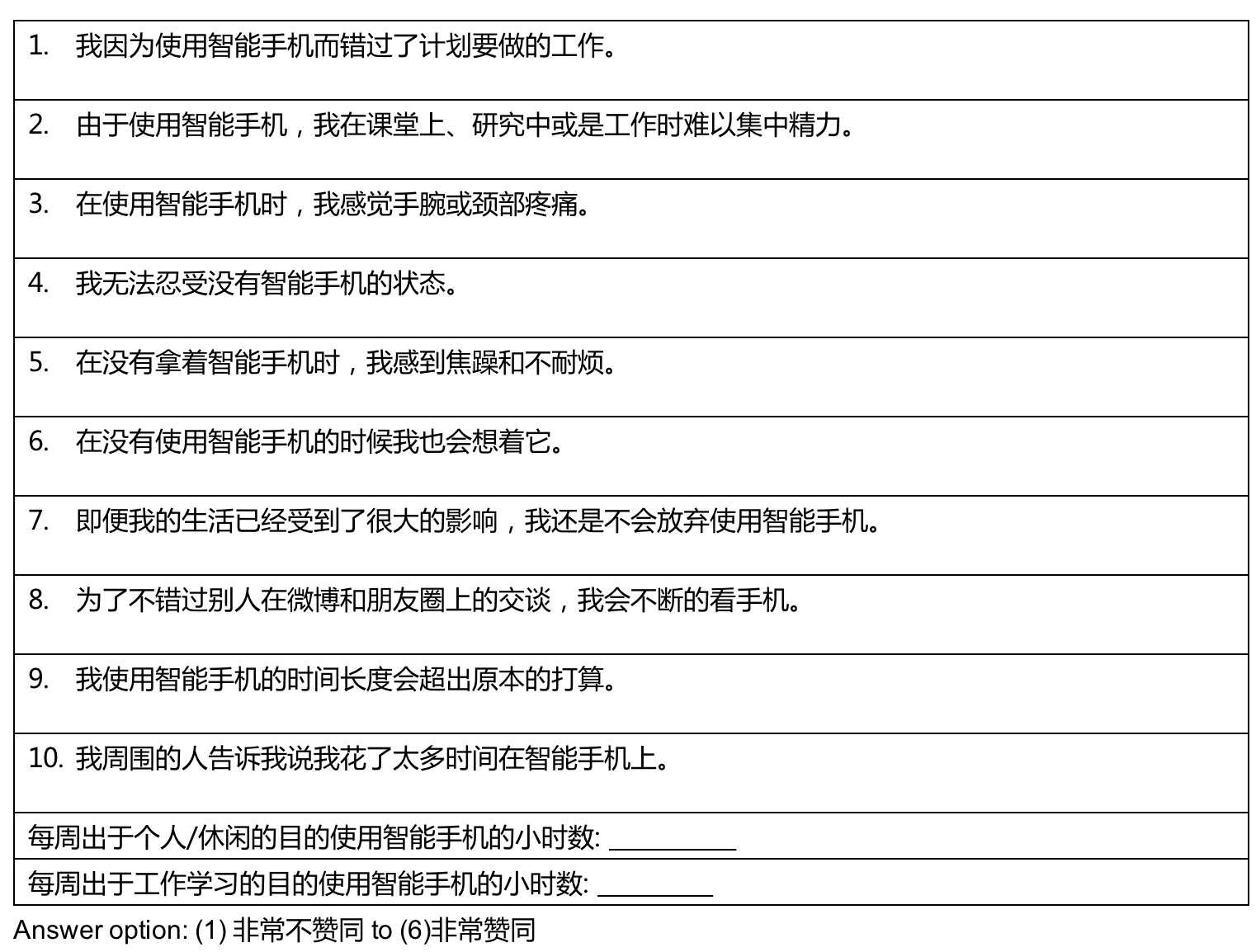


| Missing planned work due to smartphone use |
| --- |
| Having a hard time concentrating in class, while doing assignments, or while working due to smartphone use |
| Feeling pain in the wrists or at the back of the neck while using a smartphone |
| Won’t be able to stand not having a smartphone |
| Feeling impatient and fretful when I am not holding my smartphone |
| Having my smartphone in my mind even when I am not using it |
| I will never give up using my smartphone even when my daily life is already greatly affected by it. |
| Constantly checking my smartphone so as not to miss conversations between other people on Twitter or Facebook |
| Using my smartphone longer than I had intended |
| The people around me tell me that I use my smartphone too much. |
| Smartphone use in hours each week for private/leisure: _____ |
| Smartphone use in hours each week for business: ___________ |

Answer option: (1) strongly disagree to (6) strongly agree

**Table S2: Partial correlations between smartphone addiction (SAS) score and the grey matter volume (GMV) of the brain structrues**

|  | **GMV of sgACC** | **GMV of cdACC** | **GMV of pgACC** | **GMV of NAc** |
| --- | --- | --- | --- | --- |
| r | -.140 | .003 | -.039 | -.118 |
| p | .304 | .982 | .773 | .385 |

Note that no significant association between SAS and amygdala volume was observed (r = .055, p = .687)

Abbreviations: subgenual ACC (sgACC); pregenual ACC (pgACC); caudodorsal ACC; nucleus accumbens (NAc). Partial correlations are calculated with age, gender, trait anxiety and depression levels included as controlled variables.

**Table S3: Partial correlations between WeChat addiction / usage scores and the grey matter volume (GMV) of the brain structures**

| **Variable** | **WeChat Addiction** | **Texting** | **Voice Messaging** | **Paying** | **NAc** | **sgACC** | **cdACC** | **pgACC** |
| --- | --- | --- | --- | --- | --- | --- | --- | --- |
| WeChat Addiction |  | r = .20,  p = .150 | r = .48,  p < .001 | r = .26,  p = .049 | r = -.07,  p = .587 | r = -.31,  p = .021 | r = .04,  p = .789 | r = -.05,  p = .726 |
| Texting |  |  | r = .14,  p = .288 | r = .43,  p = .001 | r = -.22,  p = .098 | r = -.04,  p = .752 | r = -.08,  p = .552 | r = -.09,  p = .493 |
| Voice Messaging |  |  |  | r = .17,  p = .210 | r = -.12,  p = .384 | r = -.17,  p = .223 | r = -.15,  p = .282 | r = -.15,  p = .280 |
| Paying |  |  |  |  | r = -.32,  p = .015 | r = -.10,  p = .483 | r = -.07,  p = .610 | r = -.05,  p = .694 |
| NAc |  |  |  |  |  | r = .16  p = .250 | r = .25,  p = .066 | r = .23,  p = .085 |
| sgACC |  |  |  |  |  |  | r = .48,  p < .001 | r = .69,  p < .001 |
| cdACC |  |  |  |  |  |  |  | r = .79,  p < .001 |
| pgACC |  |  |  |  |  |  |  |  |

Partial correlations are calculated with age, gender, trait anxiety and depression levels included as controlled variables.

**Age, gender and the questionnaire data**

MANOVA revealed no significant effect of gender on all variables in Table 1. Moreover, a trend significant finding could be observed with females spending more private time on WeChat than males (F_(1,59)_ = 3.60, p = .063). Age correlated significantly with private smartphone usage (r = -.26, p = .042), WeChat addiction (r = .27, p = .035) and its subcales (loss of control, r =. 26, p =.045 and social problems, r = .27, p = .037). Finally, age was also significantly associated with sending text-messages (r = .31, p = .016).

***Smartphone Addiction and WeChat addition***

Smartphone addiction and WeChat addiction significantly correlate with each other (r = .37, p = .004). As one can see from the associations between smartphone addiction and the WeChat addiction subscales, both subscales are equally associated with smartphone addiction (WeChat loss of control: r = .35, p = .006 and WeChat social problems: r = .36, p = .005). Partializing out age, the correlations even get higher: WeChat and smartphone addiction: r = .42, p = .001; WeChat loss of control and smartphone addiction: r = .40, p =.002; WeChat social problems and smartphone addiction: r = .41, p = .001.

**References**

Kwon, M., Kim, D. J., Cho, H., & Yang, S. (2013). The smartphone addiction scale: development and validation of a short version for adolescents. *PloS one*, *8*(12), e83558.
